# Draft Genome Sequence of *Daldinia childiae* JS-1345, an Endophytic Fungus Isolated from Stem Tissue of Korean Fir

**DOI:** 10.1128/MRA.01284-19

**Published:** 2020-04-02

**Authors:** Jung A Kim, Jongbum Jeon, Sook-Young Park, Mi Jin Jeon, Joo-Hong Yeo, Yong-Hwan Lee, Soonok Kim

**Affiliations:** a Genetic Resources Assessment Division, National Institute of Biological Resources, Incheon, South Korea; b Department of Agricultural Biotechnology, Interdisciplinary Program in Agricultural Genomics, Center for Fungal Genetic Resources, Center for Fungal Pathogenesis, Seoul National University, Seoul, South Korea; c Department of Plant Medicine, Sunchon National University, Suncheon, South Korea; University of California, Riverside

## Abstract

The fungus Daldinia childiae strain JS-1345, isolated from stem tissue of *Abies koreana* (Korean fir), has shown strong anti-inflammatory activity. Here, we report the genome sequence of D. childiae JS-1345. The final assembly consisted of 133 scaffolds totaling 38,652,569 bp (G+C content, 44.07%).

## ANNOUNCEMENT

Abies koreana Wilson (Korean fir) is a shrub and an evergreen plant that is endemic in the mountain region of South Korea at an altitude of 1,000 to 1,900 m (1). It has been used as traditional medicine to treat colds, stomachache, indigestion, and rheumatic disease (2). Essential oil from A. koreana has exhibited antioxidant, anti-inflammatory, and antimicrobial activities (1, 3, 4).

Recently, we isolated one of its endophytic fungi, *Daldinia childiae* JS-1345, from stem tissue of an *A. koreana* specimen collected in 2013 from Mount Moodeung (35°06′29.6″N, 127°01′09.1″E) in Hwasoon, South Korea, according to previously reported protocols (5, 6). This strain was deposited in the Wildlife Genetic Resources Bank at the National Institute of Biological Resources (Incheon, South Korea) under accession no. NIBRGR0000180467. *D. childiae* is a widely distributed wood-inhabiting ascomycete fungus belonging to the family Hypoxylaceae and the order Xylariales (7). Although many studies of this fungus have focused on taxonomy and ecology, little is known about the bioactivity of its metabolites and its genomics. We report here the draft genome sequence of *D. childiae* strain JS-1345, an endophytic fungus isolated from *A. koreana*.

Genomic DNA was extracted from young mycelia grown in potato dextrose broth at 25°C with shaking at 120 rpm for 2 days using a DNeasy minikit (Qiagen, CA). A short-insert paired-end library was generated using a TruSeq Nano DNA sample prep kit (Illumina, CA) with a fragment size of 350 bp, and a long-insert mate pair library was generated using a Nextera mate pair library prep kit (Illumina, CA) with a fragment size of 10 kb. The PacBio sequencing library was prepared using the SMRTbell template prep kit (Pacific Biosciences, CA). Genome sequencing was performed at Theragen Etex Bio Institute in Suwon, South Korea, following the strategies described previously (8). A total of 615,019 reads with an average length of 7,965 bp were generated from 4 cells of the PacBio Sequel platform using the overlap-layout-consensus (OLC) algorithm (9). About 5.6 Gb of short sequences from a paired-end library with an average insert length of 350 bp and about 11.6 Gb of sequences with 9,026,794 high-quality reads from a mate-paired library with an average insert length of 10 kb after filtering with NextClip v1.3 (10) were generated. Short-read sequences were assembled using SOAPdenovo v2.04 (9) with a kmer value of 41. These two assemblies were merged with HaploMerger2 (11). Merged sequences were cleaned twice using the faDnaPolishing.pl script provided by HaploMerger2 (removeShortSeq=500) (11). Scaffolding and gap filling were performed using SSPACE-Standard v3.0 (12), SSPACE-LongRead v1.1 (13), and GapFiller v1.10 (14) using default parameters. To summarize, the genome reads containing the nuclear genome were assembled into 133 scaffolds (263 contigs) with a total length of 38,652,569 bp and an *N*_50_ value of 3.27 Mb from the 22.16 Gb (556× genome size) of genome sequences. Genome assembly was validated using BUSCO v3.0.2b, which showed 98.2% of the benchmarked universal single copy orthologs (BUSCOs) as complete, including 3 complete and duplicated BUSCOs, against a set of 290 fungal genes (15). The G+C content of the assembled genome was 44.07%.

Gene prediction was performed by AUGUSTUS v3.2.1 (16) and produced 10,072 protein-coding gene models, of which 9,824 had homologs in the UniProt or NCBI nr and InterPro databases. Biosynthetic gene clusters with 25 polyketide synthases and 14 nonribosomal protein synthetases were found by antiSMASH v5.0 (17, 18). We further identified 418 genes encoding transcription factor genes using the Fungal Transcription Factor Database (FTFD) v1.0 (19), 124 cytochrome P450 genes using the Fungal Cytochrome P450 Database v1.0 (20), 61 genes encoding plant cell wall-degrading enzymes using the Fungal Plant Cell Wall-Degrading Enzyme Database v1.0 (21), and 6 genes encoding laccases and 26 genes encoding peroxidases using fPoxDB v1.0 (22). This draft genome sequence will contribute to phylogenomic analyses of fungi in *Xylariales* and to the identification and functional analyses of genes involved in active compound biosynthesis.

### Data availability.

The whole-genome sequence of *D. childiae* JS-1345 obtained in this study has been deposited in GenBank under accession no. VYXO00000000. The version described in this article is the first version, VYXO01000000. PacBio and Illumina sequence data were also deposited in the SRA under accession no. SRR10154460 to SRR10154462 (BioProject no. PRJNA566056).
